# Responsiveness of the Early Childhood Oral Health Impact Scale (ECOHIS) is related to dental treatment complexity

**DOI:** 10.1186/s12955-017-0756-z

**Published:** 2017-09-20

**Authors:** Tatiane F. Novaes, Laura Regina A. Pontes, Julia G. Freitas, Carolina P. Acosta, Katia Cristina E. Andrade, Renata S. Guedes, Thiago M. Ardenghi, José Carlos P. Imparato, Mariana M. Braga, Daniela P. Raggio, Fausto M. Mendes, CARDEC collaborative group, Alessandra Reyes, Amanda F. Oliveira, Ana Estela Haddad, Ana Flavia B. Calvo, Ana Lidia Ciamponi, Andrezza S. Fonseca, Annelry C. Serra, Antonio Carlos L. Silva, Beatriz A. Bispo, Bruna P. Okamura, Bruna L. P. Moro, C R. Bresolin, Carolina P. Acosta, Caroline M. Moriyama, Claudio M. Pannuti, Daniela P. Souza, Daniela P. Raggio, Danilo A. Duarte, Edgard Michel-Crosato, Eduardo K. Kohara, Fausto M. Mendes, Gislaine Aparecida A. Dias, Haline C. M. Maia, I M P. Uribe, Isabel Cristina O. Costa, Isabela F. Martins, José Carlos P. Imparato, Juan Sebastian Lara, Judith L. Perlmuter, Julia G. Freitas, Júlia Maria R. Fonseca, Laura Regina A. Pontes, Laysa Yoshioka, Leticia T. Hashizume, Ligia Akemi, Lucas B. Gazzinelli, Lucila B. Camargo, Marcelo Bonecker, Márcia R. S. Souza, M T. Wanderley, Maria Salete N. P. Corrêa, M M. Braga, Michele B. Diniz, Pamela R. L. Almeida, R M. Samuel, Renata S. Guedes, Sergio M. Covos, Simone Cesar, Tamara K. Tedesco, Tatiane F. Novaes, Thais Gimenez, Thiago M. Ardenghi

**Affiliations:** 1 Department of Pediatric Dentistry, School of Dentistry, University of São Paulo, Av. Lineu Prestes, 2227, São Paulo, 05508-000 SP Brazil; 2 School of Dentistry, Cruzeiro do Sul University, São Paulo, Brazil; 3 School of Dentistry, Centro Universitário Franciscano, Santa Maria, Brazil; 4 Departament of Stomatology, Federal University of Santa Maria, Santa Maria, Brazil

**Keywords:** Oral health-related quality of life, Responsiveness, Preschool children, Dental treatment, Dental caries

## Abstract

**Background:**

The responsiveness of the Early Childhood Oral Health Impact Scale (ECOHIS) has varied greatly across studies; hence, we hypothesized that this discrepancy could be related to the complexity of dental treatment received. Thus, we aimed to evaluate the responsiveness of the ECOHIS to changes in oral health-related quality of life (OHRQoL) following dental treatments of varying complexity in preschool children.

**Methods:**

Preschool children aged 3 to 6 years were selected; their parents responded to the ECOHIS at baseline. The parents responded to the ECOHIS again and a global transition question 30 days after the children were treated. The type of treatment received by the children was categorized according to complexity, as follows: 1) non-operative treatment only, 2) restorative treatment, and 3) endodontic treatment and/or tooth extraction. Change scores and effect sizes (ES) were calculated for total scores, as well as considering the different treatment types and global transition question responses.

**Results:**

Of the 152 children who completed the study, the ECOHIS yielded large ES for total scores (0.89). The children showed increasing ES values associated with better perception of improvement, assessed by the global transition question. The magnitude of ES after treatment was related to treatment complexity (0.53, 0.92 and 1.43, for children who received non-operative treatment only, restorative treatment, and endodontic treatment and/or tooth extraction, respectively).

**Conclusions:**

Parents whose children required more complex dental treatment are more likely to perceive treatment-related changes to OHRQoL assessed with the ECOHIS.

**Electronic supplementary material:**

The online version of this article (doi: 10.1186/s12955-017-0756-z) contains supplementary material, which is available to authorized users.

## Background

The use of patient-centered outcome measures in several fields of healthcare research [1–3], including dentistry [4, 5], has become a subject of increasing interest over the last few years. Among this group of outcomes, oral health-related quality of life (OHRQoL) has been the most studied outcome measure across all age groups [6, 7]. In preschool children, several studies have demonstrated the impacts of different oral health problems on quality of life [8–13], and most of these studies have used the Early Childhood Oral Health Impact Scale (ECOHIS) [14] to assess OHRQoL.

The ECOHIS was developed, in English, in 2007 [14], and it was subsequently translated and adapted to several languages [15–21]. The questionnaire consists of a parental proxy report with acceptable internal consistency and reliability, as well as validity, in varying populations [8, 14, 15, 17–21]. Moreover, previous studies have investigated the responsiveness of the instrument to dental treatment [22–26], with some controversy; whilst some authors have asserted that the ECOHIS is able to measure treatment-associated changes [22, 23, 25], others have observed only modest responsiveness [24, 26].

These discrepancies are probably due to differences in study designs. While an investigation evaluating the responsiveness of the ECOHIS was conducted in a primary dental care setting [24], and thus among children presenting low-severity oral health problems, most studies were conducted in secondary and tertiary dental care settings, among children with more severe oral health problems or treated under general anesthesia [22, 23, 25, 26]. Nevertheless, these authors did not distinguish the magnitude of responsiveness according to the severity of oral health problems or complexity of dental treatment received. We hypothesize that the responsiveness of the ECOHIS varies according to dental treatment complexity; compared to patients with minor oral health problems, those with more severe oral health problems are likely to be more sensitive to improvements following dental treatment.

To test this hypothesis, the primary aim of this study was to investigate the ability of the ECOHIS to detect changes in OHRQoL, 30 days after dental treatments of varying complexities, in preschool children. The secondary aim was to explore whether the change scores, occurring over shorter and longer periods (7 days and 90 days, respectively) after treatment, were similar to those obtained with the ECOHIS after 30 days. This additional evaluation was conducted as assessment periods differ substantially between studies [22–26].

## Methods

### Design, participants, and ethical considerations

This longitudinal study, designed to assess the responsiveness of the ECOHIS, was conducted among preschool children who had sought assistance from our dental school. The protocol was approved by the Local Committee for Ethics in Research, and informed consent was provided by guardians prior to their children’s participation in the study.

The present research was nested within a clinical trial, the CARies DEtection in Children (CARDEC 1) trial [27]. Briefly, the aim of this trial was to compare different diagnostic strategies for the detection of non-evident caries lesions in preschool children. Children were assessed using visual inspection only or by a combination of visual inspection and radiography. All children were treated with specific protocols, and several outcomes were evaluated for 2 years. The research protocol is registered at Clinicaltrials.gov (NCT02078453), and published elsewhere [27].

To assess ECOHIS responsiveness, a sample size calculation was performed based on a significance level of 5% and statistical power of 80%, using a parametric approach for paired samples (paired t-test). The minimal important difference (MID) was estimated considering an effect size (ES) of 0.5 and standard deviation (SD) of change scores around 4.0 [26]. Following these parameters, 126 participants were required. As change scores obtained in several studies using the ECOHIS have not been normally distributed, we added 15% to this number to avoid loss of statistical power [28]. Thus, an estimated minimum sample size of 145 children were required for this study.

The first 160 children (an addition of 10% to deal with dropouts), included in the main clinical trial, were invited to participate in the study.

### Inclusion and exclusion criteria and study procedures

The inclusion criteria were the same as those in the main clinical trial: (i) preschool children who sought dental assistance from our dental school, (ii) children aged 3 to 6 years, and (iii) children with at least one primary molar. Children whose parents refused to participate in the study and children who presented behavioral problems during the first appointments were excluded.

The included children were assessed by two calibrated examiners, using visual inspection only or visual inspection and radiography. Visual inspection was performed using the International Caries Detection and Assessment System (ICDAS), and activity of caries lesions was assessed based on the clinical features of the lesion [29]. For children submitted to radiographic examination, conventional bitewing radiographies were taken. For all children, independent of the experimental group, periapical radiographies were taken of teeth with extensive caries lesions, to decide if endodontic treatment or extraction were required. After the diagnostic procedures, the examiners prescribed individual treatment plans based on predetermined protocols [27]. These protocols included: oral hygiene using fluoride toothpaste (1000–1500 ppm) and dietary advice, non-operative treatment, operative treatment, endodontic treatment and/or dental extraction. More details on the treatments performed are provided in a previous publication [27].

At the first appointment, independent of the dental examination, the children’s parents answered the Brazilian version [15, 21] of the ECOHIS [14]. The questionnaire consists of 13 questions, divided into two sections: a child impact section with 4 domains (child symptoms, function, psychological, and self-image/social interaction domains) and a family impact section with 2 domains (parental distress and family function). Respondents answer the questions using a rating scale from 0 to 5, where: 0 = never, 1 = hardly ever, 2 = occasionally, 3 = often, 4 = very often, and 5 = do not know. Total scores can range from 0 to 52, with higher scores indicative of greater negative impacts of oral health problems on quality of life [14]. Answers assigned a score of 5 (do not know) or missing answers were both treated as missing items. When respondents had up to two missing items in the child impact section or up to one missing item in the family section, the mean of the remaining items in that section were used to impute values for missing items. Questionnaires with more than two missing items in the children impact section and more than one missing item in the family impact section were excluded due to improper answering [14].

Subsequently, the children were treated, and 7, 30, and 90 days after treatment, the same parent completed the ECOHIS for a second time. The interviewer was unaware of the previous answers obtained, or the treatment received by the children, and the respondents did not have access to their baseline responses. Parents also responded to a global transition question on the changes they perceived in their children’s oral health after treatment; possible answers included: worsened a lot, worsened a little, no changes observed, improved a little, and improved a lot.

### Variables and data analysis

Prior to statistical comparison, to evaluate normality and homogeneity of variance, all quantitative outcome variables were submitted to Kolmogorov-Smirnov and Levene’s tests. As no measures were normally or homogeneously distributed, we used non-parametric tests. We conducted between-group comparisons (visual inspection only vs visual inspection and radiographic examination) of ECOHIS scores at baseline and after treatment using Mann-Whitney tests.

To assess internal responsiveness (distribution-based approach), we compared domain and total ECOHIS scores obtained at baseline and 30 days after treatment using Wilcoxon tests. We also calculated change scores and ES; the latter were estimated by dividing the mean of the differences by the SD of the baseline scores.

We assessed external responsiveness (anchor-based approach) by comparing ECOHIS scores obtained at baseline and after treatment according to global transition question responses, using Wilcoxon tests. Change scores were also calculated and compared according to different transition ratings, using Kruskal-Wallis tests.

We calculated MIDs using both distribution-based and anchor-based approaches [30]. In the distribution-based approach, the MID was assumed by calculating one-half of the SD of the total ECOHIS scores at baseline [30]. The mean ECOHIS change scores, obtained from parents who answered that their children ‘improved a little’ after dental treatment, were considered the MID derived through the anchor-based procedure [24, 30].

We also performed regression analyses to investigate the influence of treatment type on change scores. The types of treatment required were classified as follows:

- Non-operative treatment only: These children presented either no caries lesions, or lesions that did not require operative treatment. Non-operative treatment was conducted via oral hygiene and dietary advice, orientation for use of fluoride toothpaste (fluoride concentration of 1.000 to 1.500 ppm) and varnish.
- Restorative treatment: In addition to non-operative treatment, these children had cavitated lesions, and were treated with Atraumatic Restorative Treatment for shallower caries lesions, and indirect pulp capping for deeper lesions.
- Endodontic treatment or tooth extraction: These children presented teeth with pulp involvement and/or teeth that required extraction, either due to caries or dental injuries.

When children required different types of treatment (e.g. some teeth required restorative treatment and others endodontic treatment), they were classified according to the most severe treatment.

We explored other independent variables, including: sex (male or female), age (3 to 4 years vs. 5 to 6 years), caries experience (i.e. the number of decayed, missing, and filled surfaces of primary teeth; dmf-s < 4 vs dmf-s ≥ 4), and global transition question responses (i.e. no change, improved a little, or improved a lot). For these analyses, two outcome variables were considered. First, using the MID values obtained from both anchor- and distribution- based methods, children were dichotomized into those scoring below the MID and those scoring at least the MID (or above). To evaluate the influence of independent variables on this outcome, we used univariate and multiple Poisson regression analyses with robust variance; relative risk (RR) values and 95% confidence intervals (95% CIs) were calculated. Multiple models were built, partly based on statistical significance, but also including some important, but not statistically significant, variables. Second, associations with entire change scores (a quantitative discrete variable) were analyzed with Poisson regression analyses with robust variance; ECOHIS ratio scores (RS) and 95% CIs were calculated. As some children presented negative differences after treatment, data was transformed to positive entire values for analytical purposes; but it was subsequently back-transformed for presentation purposes.

Finally, change scores and ES were calculated, according to ECOHIS domains and total scores obtained at baseline and 7, 30, and 90 days after treatment. Comparisons between change scores at different time points were made using Friedman tests.

All analyses were carried out using Stata 13.0 (Stata Corp, College Station, TX, USA) and MedCalc 13.1.2.0 (MedCalc Software bvba, Ostend, Belgium). The level of significance was fixed at 5%.

## Results

Of the 160 children invited to participate in the study, 152 were followed-up for 30 days after treatment (positive follow-up rate of 95.0%). The reasons for dropout were: children with inadequate behavior during the treatment appointments (*n* = 4), children who failed to attend the treatment appointments (*n* = 2), and children who were excluded due to improper ECOHIS responses (*n* = 2).

There were no significant differences in baseline ECOHIS scores according to the groups of the clinical trial . Therefore, we did not include this variable in the subsequent analyses.

Seventy-six (50.0%) children were male, 70 (46.1%) were aged 3 to 4 years, 82 (53.9%) were aged 5 to 6 years, and 72 (47.4%) presented with dmf-s < 4. Thirty-five (23.0%) children required non-operative treatment only, 83 (54.6%) required restorative treatment, and 34 (22.4%) required endodontic treatment and/or tooth extractions. The mean (SD) dmf-s was 8.0 (10.5), with a range of 0 to 54.

The ECOHIS domain and total scores obtained 30 days after treatment were significantly lower than at baseline (Table 1). The ES were large (≥ 0.8) or moderate (0.3–0.7) for the different domains, and large for the total scores (0.89) (Table 1).

**Table 1:** Total and individual domains scores, change scores and effect sizes obtained with the Early Childhood Oral Health Impact Scale (ECOHIS) at the baseline, and 30 days after finishing the dental treatment (*n* = 152)

Time after treatment		Baseline	30 days	Change scores	Effect size
ECOHIS	Possible ranges	Mean (SD)	Mean (SD)	Mean (SD)	
Child section	0–36				
Symptoms (1 item)	0–4	1.15 (1.20)	0.16 (0.49)^*^	0.99 (1.26)	0.83
Function (4 items)	0–16	2.14 (2.81)	0.15 (0.73)^*^	1.99 (2.87)	0.70
Psychological (2 items)	0–8	1.17 (1.77)	0.03 (0.24)^*^	1.14 (1.79)	0.64
Self-image/social interaction (2 items)	0–8	0.59 (1.35)	0.01 (0.08)^*^	0.59 (1.33)	0.43
Family section	0–16				
Parent distress (2 items)	0–8	1.91 (2.36)	0.05 (0.44)^*^	1.86 (2.41)	0.79
Family function (2 items)	0–8	1.01 (1.55)	0.03 (0.33)^*^	0.98 (1.56)	0.63
Total ECOHIS scores	0–52	7.97 (8.46)	0.43 (1.68)^*^	7.54 (8.56)	0.89

*SD* standard deviation

^*^Statistically significant difference between the ECOHIS scores at the baseline and 30 days after finishing the treatment (*p* < 0.05, calculated by Wilcoxon test)

Regarding global transition question responses, no parents responded that their children ‘worsened a lot’ or ‘worsened a little’ after treatment. There was no significant difference between the scores obtained at baseline and follow up in those who perceived ‘no changes’ after treatment. However, those who ‘improved a little’ or ‘improved a lot’ had significantly lower ECOHIS scores after treatment (Table 2). Larger ES were observed with greater improvements. Moreover, the change scores for children that ‘improved a lot’ were significantly higher than for children who ‘improved a little’ or did not change. Conversely, no difference was observed between children who ‘improved a little’ or did not change (Table 2).

**Table 2:** The Early Childhood Oral Health Impact Scale (ECOHIS) scores at the baseline and 30 days after the treatment, and mean change scores by global transition ratings

Global transition ratings	N	ECOHIS scores Mean (SD)		*p* ^a^	Change scores	ES
		Baseline	After treatment		Mean (SD)	
After 30 days						
No change	20	4.7 (7.2)	1.6 (4.1)	0.110	3.1 (7.8)^a^	0.43
Improved a little	22	5.5 (7.0)	0.6 (1.0)	< 0.001	4.9 (6.5)^a^	0.70
Improved a lot	110	9.1 (8.7)	0.2 (0.7)	< 0.001	8.9 (8.7)^b^	1.02

*SD* Standard deviation, *ES* Effect size

Different letters indicate statistical significant differences among children according different global transition ratings (p < 0.05, by Kruskal-Wallis test)

^a^calculated with Wilcoxon test

The calculated MIDs were 4.2 and 4.9, using the distribution- and anchor- based approaches, respectively. Therefore, we observed a consistency in both MID values. Children who presented change scores ≥5.0 after treatment were classified as reaching the MID of the ECOHIS.

In our sample, 77 (50.7%) children scored at least the MID. Table 3 provides the frequency of children scoring at least the MID according to the independent variables, change scores, and ES. The highest ES were observed in children requiring endodontic treatment and/or tooth extraction, followed by children with ≥4 dmf-s (Table 3).

**Table 3:** Children scoring below Minimal Important Difference (MID) and change scores for differences among scores of the Early Childhood Oral health Impact Scale (ECOHIS) at the baseline and 30 days after finishing the dental treatment considering some independent variables (*n* = 152)

Independent variables	Children scoring at least the MID		Change scores	ES
	No (%)	Yes (%)	Mean (SD)	
Sex				
Male	38 (50.0)	38 (50.0)	8.2 (9.3)	0.89
Female	37 (48.7)	39 (51.3)	6.8 (7.8)	0.90
Age				
3 to 4 years old	40 (57.1)	30 (42.9)	6.5 (8.2)	0.81
5 to 6 years old	35 (42.7)	47 (57.3)	8.4 (8.8)	0.96
dmf-s				
< 4	57 (79.2)	15 (20.8)	2.7 (4.3)	0.63
≥ 4	18 (22.5)	62 (77.5)	11.9 (9.1)	1.36
Treatment need				
Only non-operative treatment	29 (82.9)	6 (17.1)	2.3 (4.5)	0.53
Restorative treatment	37 (44.6)	46 (55.4)	8.2 (0.2)	0.92
Endodontic treatment /tooth extraction	9 (26.5)	25 (73.5)	11.3 (7.8)	1.43
Global transition ratings				
No change	16 (80.0)	4 (20.0)	3.1 (7.8)	0.43
Improved a little	14 (63.6)	8 (36.4)	4.9 (6.5)	0.70
Improved a lot	45 (40.9)	65 (59.1)	8.9 (8.7)	1.02

dmf-s = Number of decayed, missed or filled surfaces of primary teeth

MID = minimal important difference (Change scores ≥5)

*SD* Standard deviation, *ES* Effect Size

Multiple Poisson regression analyses revealed that children requiring restorative treatment and endodontic treatment and/or tooth extraction were more likely to score at least the MID, compared to children requiring non-operative treatment only (Table 4). Furthermore, a global transition question response of ‘improved a lot’ presented significant associations. Higher ECOHIS scores at baseline were associated with a higher probability of reaching at least the MID. Sex and age were not significantly associated with any outcome in either the univariate or multiple analyses (Table 4). As type of treatment and caries experience at baseline are collinear variables, an alternative final model including caries experience instead of dental treatment was built. In this final model, children with dmf-s ≥ 4 presented a RR (95% CI) of 2.10 (1.30 to 3.43; *p* = 0.003), adjusted for sex, age, baseline total ECOHIS scores, and global transition question responses.

**Table 4:** Univariate and multiple Poisson analysis to evaluate the association of children scoring below Minimal Important Difference (MID) and change scores for differences among scores of the Early Childhood Oral health Impact Scale (ECOHIS) at the baseline and 30 days after finishing the dental treatment (outcome variables) and some independent variables (*n* = 152)

Independent variables	Children scoring below MID		Change scores	
	Unadjusted RR	Adjusted RR	Unadjusted RS	Adjusted RS
Sex (ref.: Male)				
Female	1.03 (0.75 to 1.41)	1.26 (0.97 to 1.63)	0.91 (0.77 to 1.09)	1.03 (0.98 to 1.08)
Age (ref.: 3 to 4 yrs. old)				
5 to 6 years old	1.34 (0.96 to 1.86)	1.13 (0.86 to 1.47)	1.13 (0.95 to 1.35)	1.00 (0.95 to 1.06)
dmf-s (ref.: < 4)		^a^		^a^
≥ 4	3.72^*^ (2.33 to 5.93)		1.85^*^ (1.62 to 2.13)	
Treatment need (ref.: only non-operative)				
Restorative treatment	3.23^*^ (1.52 to 6.88)	2.02^*^ (1.03 to 3.96)	1.57^*^ (1.30 to 1.90)	1.07 (0.99 to 1.16)
Endodontic treatment /tooth extraction	4.29^*^ (2.01 to 9.16)	2.15^*^ (1.07 to 4.35)	1.87^*^ (1.54 to 2.27)	1.13^*^ (1.03 to 1.24)
Global transition ratings (ref.: No change)				
Improved a little	1.82 (0.64 to 5.14)	1.64 (0.73 to 3.68)	1.16 (0.80 to 1.67)	1.10 (0.93 to 1.31)
Improved a lot	2.95^*^ (1.21 to 7.21)	2.14^*^ (1.05 to 4.38)	1.52^*^ (1.11 to 2.09)	1.18 (1.00 to 1.38)
Total ECOHIS score at baseline (quant. var.)	1.07^*^ (1.05 to 1.08)	1.06^*^ (1.04 to 1.07)	1.05^*^ (1.04 to 1.06)	1.05^*^ (1.04 to 1.05)

dmf-s = Number of decayed, missed or filled surfaces of primary teeth

MID = minimal important difference (Change scores ≥5)

*RR* Relative risk, *RS* ECOHIS ratio scores. Figures in parenthesis ate 95% Confidence Intervals

^*^Association statistically significant (p < 0.05)

^a^Variable not included in the final multiple model

Children requiring endodontic treatment and/or tooth extraction presented significantly higher change scores than those requiring non-operative treatment only, but children requiring restorative treatment did not present significant differences. Another variable significantly associated with the outcomes in the final model was baseline total ECOHIS score (Table 4). Sex, age, and global transition question responses were not significant associated with ECOHIS change scores in either univariate or multiple analyses (Table 4). An alternative multiple model revealed that children with dmf-s ≥ 4 presented with significantly higher change scores than those with dmf-s < 4 (RS = 1.09; 95% CI = 1.01 to 1.19, *p* = 0.035, adjusted for sex, age, global transition questionnaire responses, and baseline total ECOHIS scores).

We observed that change scores and ES obtained 7 and 30 days after treatment were very similar, with no significant differences (Table 5). However, after 90 days, values were slightly lower than after 30 days. In most domain and in total scores, we did not observe any significant differences. The exception was the Function domain; significantly lower change scores were observed after 90 days, compared to after 7 or 30 days (Table 5).

**Table 5:** Mean of change scores of the Early Childhood Oral Health Impact Scale (ECOHIS) at the baseline and after the treatment collected in different times after finishing the treatment and effect sizes

Change scores^*^	7 days (*n* = 141)		30 days (*n* = 152)		90 days (*n* = 147)	
	Mean (SD)	ES	Mean (SD)	ES	Mean (SD)	ES
Child section						
Symptoms	0.98 (1.24) ^a^	0.83	0.99 (1.26)^a^	0.83	0.82 (1.28)^a^	0.68
Function	2.01 (2.87) ^a^	0.72	1.99 (2.87)^a^	0.70	1.67 (2.67)^b^	0.58
Psychological	1.05 (1.73) ^a^	0.62	1.14 (1.79)^a^	0.64	1.06 (0.74)^a^	0.60
Self-image/social interaction	0.57 (1.37) ^a^	0.42	0.59 (1.33)^a^	0.43	0.54 (1.31)^a^	0.39
Family section						
Parent distress	1.87 (2.35) ^a^	0.80	1.86 (2.41)^a^	0.79	1.84 (2.31)^a^	0.79
Family function	1.06 (1.60) ^a^	0.66	0.98 (1.56)^a^	0.63	1.05 (1.59)^a^	0.66
Total ECOHIS scores	7.55 (8.47) ^a^	0.90	7.54 (8.56)^a^	0.89	6.97 (7.88)^a^	0.82

Different letters indicate statistical significant differences among changes scores considering different times after the treatment (*p* < 0.05, through Friedman test)

*SD* Standard deviation, *ES* Effect size

^*^Changes in the ECOHIS scores obtained at the baseline and in different times after finishing the treatment

## Discussion

Responsiveness is one of the most important properties of instruments measuring the impact of health problems on patient quality of life [31]. The ECOHIS is the most widely used questionnaire for assessing OHRQoL in preschool children [8–12, 14, 15, 18, 19]. It has demonstrated responsiveness to dental treatment in different studies, albeit of different magnitudes [22–26]. We speculated that these differences were related to the complexity of dental treatment received by the patients. We found that children submitted to restorative treatment had higher positive responses to dental treatment than those submitted to non-operative treatment only. Moreover, children requiring endodontic treatment and/or tooth extraction had even higher responses. Therefore, we accepted our working hypothesis; this is the first study to associate the responsiveness of the ECOHIS with the type of dental treatment received.

We found a large ES for total ECOHIS scores, compatible with previous papers [22, 23, 25, 32] despite these studies finding higher values. It is important to highlight that participants in these previous studies were treated under general anesthesia [23, 25, 32], or had high-severity oral health problems [22]. Other authors have asserted that the responsiveness of the ECOHIS is low in populations with low-severity oral health problems, with ES varying from 0.15 to 0.30 [24, 26]. When we only considered children receiving more complex treatment, the ES (1.43) was similar to those obtained in previous studies in children with high-severity oral health problems [22, 23, 25, 32]. Nevertheless, the ES for children with low-severity oral health problems was higher (0.53) than those observed in other studies in children with low-severity oral health problems [24, 26]. These differences may be explained by the fact that our participants were children who sought dental assistance. Therefore, their parents may have been more sensitive to dental treatment-associated changes than those in previous studies.

Thus, in the present study, we clarified one of the possible reasons for the discrepancy observed among previous studies that have investigated the responsiveness of the ECOHIS [22–26, 32]: responsiveness is related to the dental treatment required, which is in turn associated with the severity of oral health problems. We found an increasing ES associated with caries severity, observed for different domain and total ECOHIS scores. We tested this association, considering the entire difference values and a dichotomous outcome. For this latter outcome, we calculated the MID [30] and children who reached at least this value. For both outcomes, the influences of dental treatment type and caries severity were explored. Moreover, the MID calculated by different distribution- and anchor- based approaches were similar (4.2 and 4.9, respectively). Therefore, the MID in the ECOHIS for preschool children after dental treatment is estimated to be around 5 scale points. The value obtained in previous research was slightly smaller (3.1), but this study was performed in a primary care setting [24].

The ECOHIS also showed external responsiveness. The global transition question responses were related to the ES obtained; responses of ‘no changes observed’ after dental treatment were associated with a lower ES. However, children who ‘improved a lot’ after treatment, had change scores that were three-times higher than children with ‘no changes’. Another variable positively associated with improvement in quality of life was baseline ECOHIS score. Higher ECOHIS scores were associated with a higher number of children reaching the MID, as well as higher change scores.

All these findings are consistent with our hypothesis. Parents usually feel guilty about oral health problems in their children, and this feeling is more common in children with higher-severity caries [33]. Whilst, there is an ECOHIS domain regarding family impact, and family feelings are translated in baseline ECOHIS responses, much of the questionnaire is dedicated to child symptoms and function. Moreover, answers are parents’ proxy reports, and children with more severe oral health conditions require higher complexity dental treatments. Taken together, these points could explain our findings; it is understandable that parents whose children present major oral health problems will be more satisfied with the dental treatment received.

Regarding our secondary aim, we evaluated responsiveness at different times after treatment. We sought to evaluate this issue as, in the current literature, assessment times after treatment are too diverse to draw robust conclusions, ranging from 7 days [22] to almost 1 year [24]. We observed a similarity when the ECOHIS was applied 7 and 30 days after treatment. However, a slight decrease in the effect was observed after 90 days. This decrease could be explained by treatment failures, or an expected temporal decrease in the perception of dental treatment importance. Interestingly, the study that observed the smallest effect of treatment via ECOHIS scores applied the questionnaire almost 1 year after treatment [24]. However, a limitation of our study is that we did not evaluate the scores after longer periods. Therefore, we can only speculate as to the causes of differences obtained across studies. Other probable explanations may include different populations, settings, or dental treatment conditions. Further studies are required to investigate these issues.

Another limitation of our study was the use of parental proxy reports of children’s OHRQoL. Some authors have asserted that parents are not always able to perceive their children’s OHRQoL accurately, with different parent perceptions compared to children self-reports. Therefore, similar research, testing questionnaires that consider both children’s and parents’ reports of OHRQoL, such as the Scale of Oral Health Outcomes for 5-year-old children (SOHO-5) [34], are necessary to ascertain whether the same pattern of responsiveness, observed in the present study with the ECOHIS, are replicated.

The strength of our study is that we demonstrated the responsiveness of the ECOHIS in relation to severity of oral health problems, or complexity of dental treatment received. Several studies have confirmed that the ECOHIS is able to detect changes related to dental treatment [22–26], and more recently, the ECOHIS was found to be sensitive to perceived oral health impairment [35]. Therefore, the ECOHIS could be a valuable tool for measuring important patient-centered outcomes in clinical trials testing various dental treatments. However, the authors should individualize MID estimation according to the complexity of treatment interventions tested in future clinical trials.

## Conclusions

Parents of children who require highly complex dental treatments are more likely to perceive treatment-related changes related to OHRQoL, measured via the ECOHIS. These changes are very similar 7 and 30 days after dental treatment; however, 90 days after treatment, the perception of improvement tends to decrease slightly.

## Additional files

Additional file 1: Persons who participate in the CARDEC collaborative group and their respectiveroles. (PDF 11 kb)

Additional file 2: Datasheet used in the analysis. (XLSX 52 kb)

## Abbreviations

95%CI: 95% confidence interval
CARDEC: CARies DEtection in Children
dmf-s: Decayed, missed and filled surfaces of primary teeth
ECOHIS: Early Childhood Oral Health Impact Scale
ES: Effect size
ICDAS: International Caries Detection and Assessment System
MID: Minimal important difference
OHRQoL: Oral health-related quality of life
RR: Relative risk
RS: ECOHIS ratio scores
SD: Standard deviation
