# Supplementary material for: Responsiveness of the Early Childhood Oral Health Impact Scale (ECOHIS) is related to dental treatment complexity
Source: Health Qual Life Outcomes. 2017 Sep 20;15:182. doi: 10.1186/s12955-017-0756-z (PMC5608161; doi:10.1186/s12955-017-0756-z)
Supplement: Supplementary file 1 — Persons who participate in the CARDEC collaborative group and their respectiveroles. (PDF 11 kb) [file 12955_2017_756_MOESM1_ESM.pdf]

***CARies DEtection in Children (CARDEC) collaborative group**

School of Dentistry, University of São Paulo, São Paulo, Brazil

Amanda Ferreira de Oliveira (dental hygienist)

Ana Estela Haddad (researcher)

Ana Lidia Ciamponi (researcher)

Andrezza Stewien Fonseca (dentist)

Annelry Costa Serra (secretary)

Antonio Carlos Lopes Silva (technician)

Beatriz de Albuquerque Bispo (undergraduate student)

Bruna de Paula Okamura (undergraduate student)

Bruna Lorena Pereira Moro (graduate student)

Carmela Rampazzo Bresolin (PhD student)

Carolina de Picoli Acosta (undergraduate student)

Caroline Moraes Moriyama (PhD student)

Claudio Mendes Pannuti (researcher)

Daniela Pereira de Souza (undergraduate student)

Daniela Prócida Raggio (researcher)

Edgard Michel-Crosato (researcher)

Eduardo Kazuo Kohara (PhD student)

Fausto Medeiros Mendes (researcher)

Gislaine Aparecida Almeida Dias (undergraduate student)

Haline Cunha Medeiros Maia (dentist)

Isaac Murisi Pedroza Uribe (graduate student)

Isabel Cristina Olegário da Costa (PhD student)

Isabela Floriano (PhD student)

José Carlos P. Imparato (researcher)

Juan Sebastian Lara (PhD student)

Julia Gomes Freitas (undergraduate student)

Laura Regina Antunes Pontes (dentist)

Laysa Yoshioka (graduate student)

Leticia Tiemi Hashizume (undergraduate student)

Ligia Akemi (dentist)

Lucas Botelho Gazzinelli (dentist)

Marcelo Bonecker (researcher)

Marcia Turolla Wanderley (researcher)

Márcia Rodrigues da Silva Souza (dental hygienist)

Maria Salete Nahás Pires Corrêa (researcher)

Mariana Minatel Braga (researcher)

Pamela Rocha Lopes de Almeida (undergraduate student)

Renata Saraiva Guedes (PhD student)

Simone Cesar (dentist)

Thais Gimenez (researcher)

School of Dentistry, Universidade Cruzeiro do Sul, São Paulo, Brazil

Danilo Antonio Duarte (researcher)

Katia Crisrina Esquivel Andrade (graduate student)

Michele Baffi Diniz (researcher)

Renata Marques Samuel (graduate student)

Tatiane Fernandes Novaes (researcher)

School of Dentistry, Universidade Paulista, Campinas, Brazil

Ana Flavia Bissoto Calvo (researcher)

Lucila Basto Camargo (researcher)

Tamara Kerber Tedesco (researcher)

School of Dentistry, Centro Universitário Hermínio Ometto, Araras, Brazil

Alessandra Reyes

School of Dentistry, Federal University of Santa Maria, Santa Maria, Brazil

Thiago Machado Ardenghi (researcher)

School of Dentistry, Universidad de la República, Montevideo, Uruguay

Judith Liberman Perlmuter (researcher)

Private office

Sergio Marcelino Covos (graphic designer)
